# Supplementary material for: Tunable Synthesis of Predominant Semi-Ionic and Covalent Fluorine Bonding States on a Graphene Surface
Source: Nanomaterials (Basel). 2021 Apr 7;11(4):942. doi: 10.3390/nano11040942 (PMC8067876; doi:10.3390/nano11040942)
Supplement: Supplementary file 1 [file nanomaterials-11-00942-s001.pdf]

nanomaterials

Supplementary Materials

Tunable Synthesis of Predominant Semi-Ionic and Covalent Fluorine Bonding States on a Graphene Surface

Jae Won Lee [1,2], Seung Pil Jeong [1], Nam-Ho You [1] and Sook Young Moon [1,*]

[1] Institute of Advanced Composite Materials, Korea Institute of Science and Technology (KIST), Chudong-ro 92, Bongdong-eup, Wanju-gun, Jeonbuk 55324, Korea; 092120@kist.re.kr (J.W.L.); jsp0103@kist.re.kr (S.P.J.); polymer@kist.re.kr (N.-H.Y.)

[2] Department of Advanced Materials Science and Engineering, Hanyang University, Ansan 15588, Korea

* Correspondence: moon.sookyoung@kist.re.kr

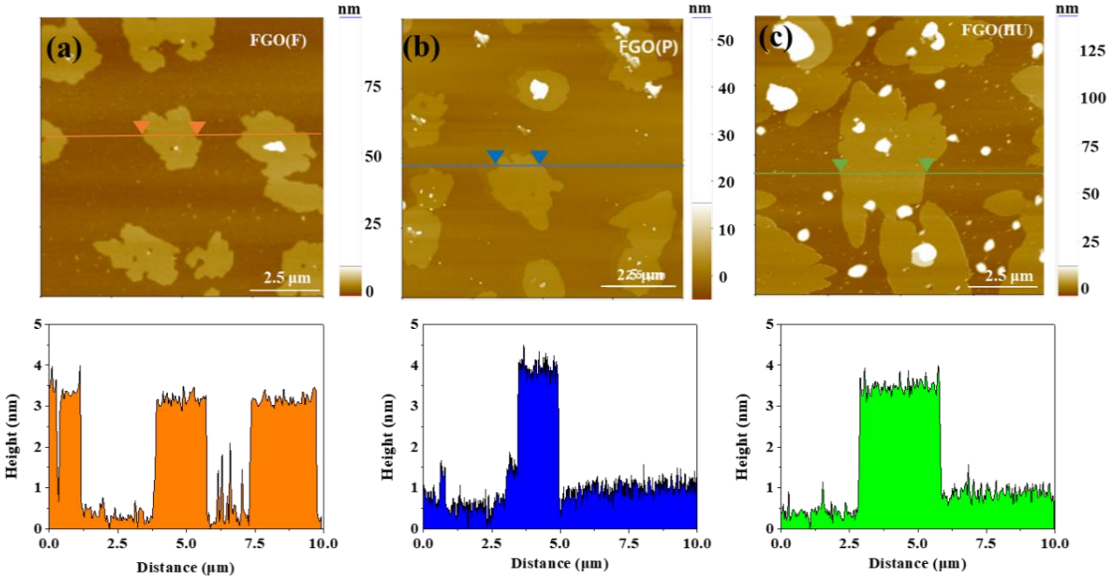

Figure S1. Morphology of all of the GOs by AFM: (**a**) FG(F), (**b**) FG(P), and (**c**) FG(HU).

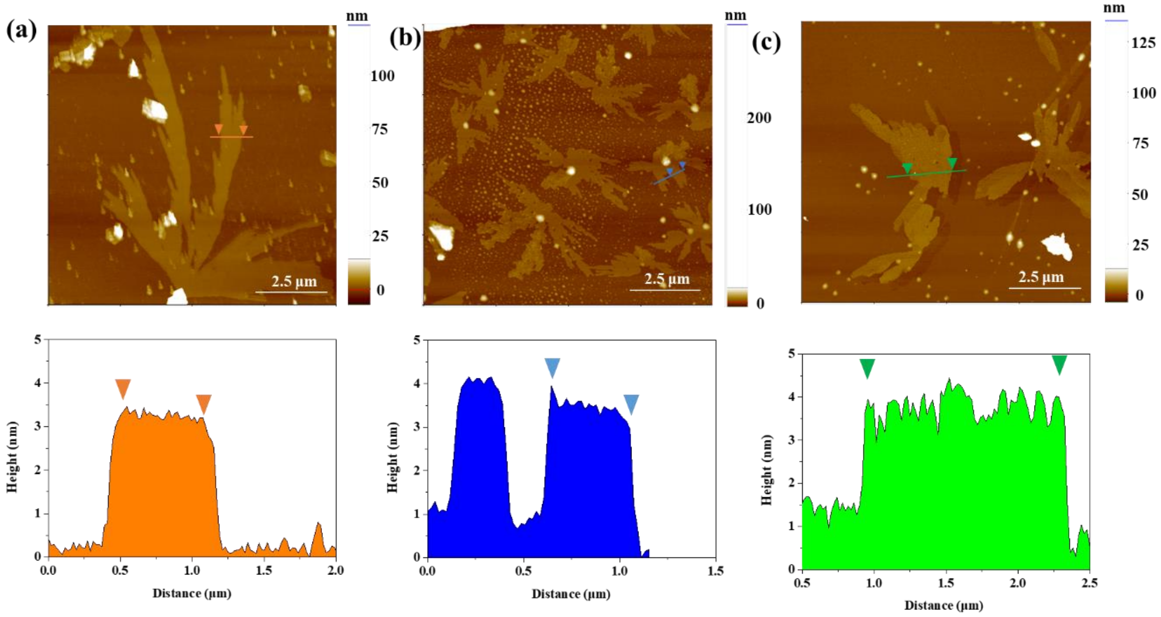

Figure S2. Morphology of all of the GOs by AFM: (**a**) GO(F), (**b**) GO(P), and (**c**) GO(HU).

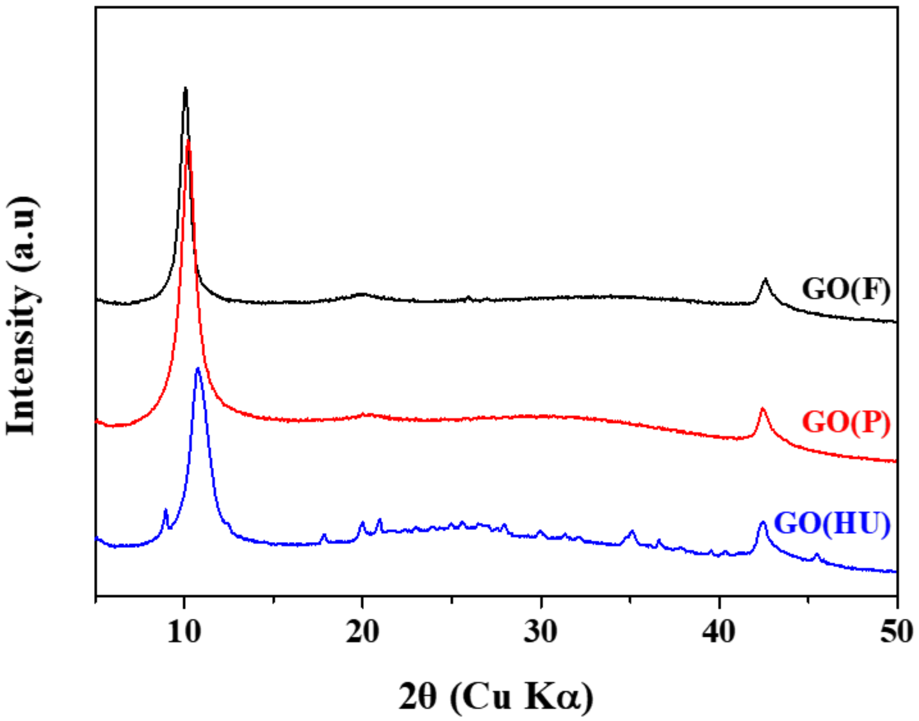

Figure S3. X-ray diffraction pattern of GOs.

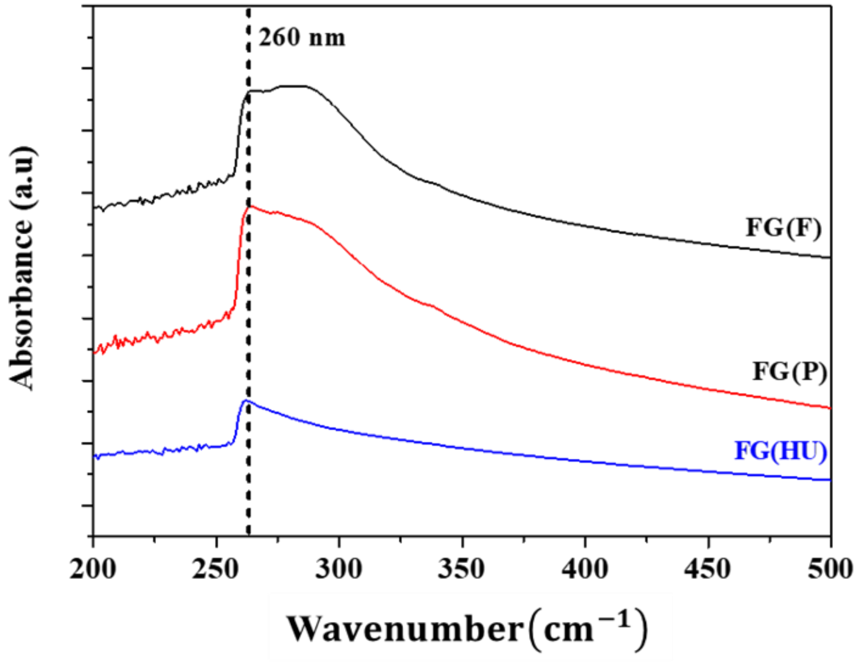

Figure S4. UV-vis spectra of FGs.

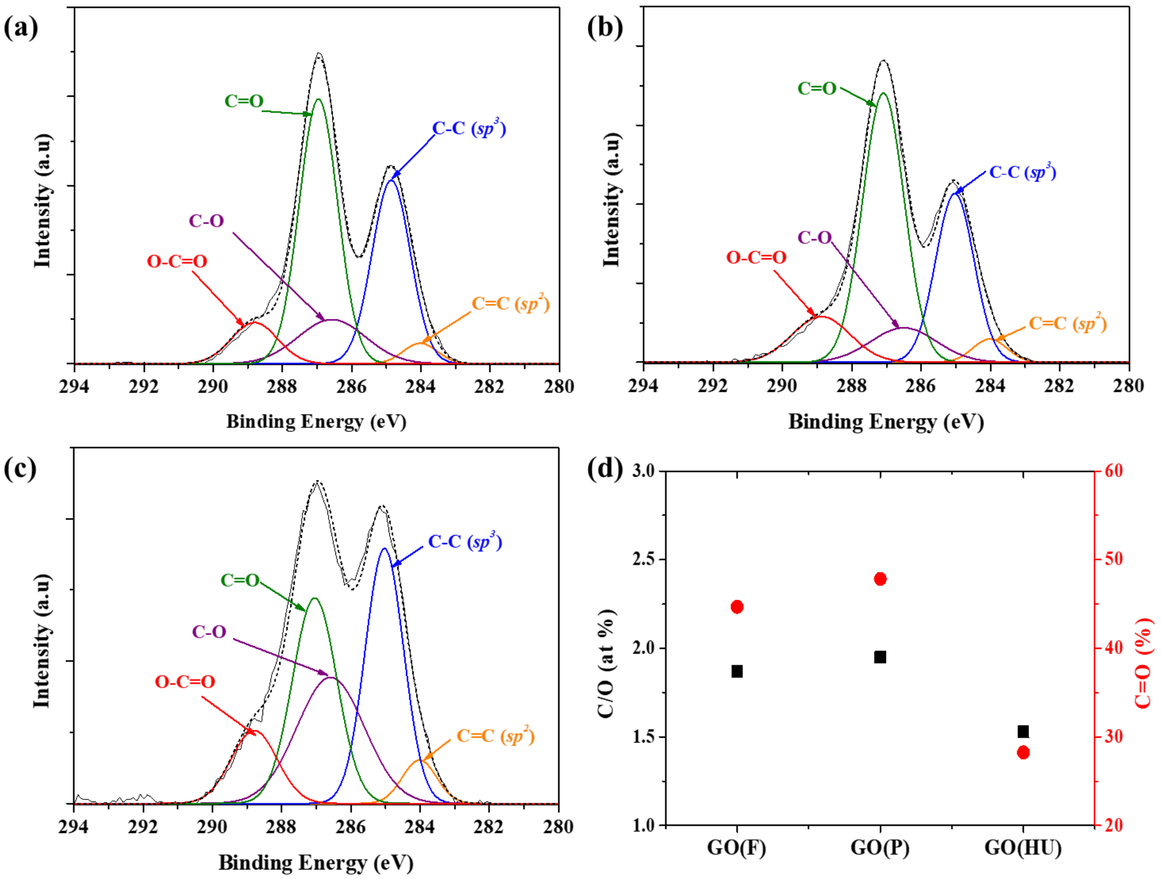

Figure S5. XPS spectra of C 1s of GO: (**a**) GO(F), (**b**) GO(P), (**c**) GO(HU), and (**d**) ratio of C/O and O=C (%).
